# On Psora

**Published:** 1802-06

**Authors:** 


### *On PSORA.*

OBSERVING in your valuable Journal, Mr. Ring's Observations on that cutanous Eruption, termed Psora, induced me to make a few remarks on the same subject, having experienced, in many instances, the ill effects of the external application of corrosive sublimate in this, as also *many other* cutaneous affections, by frequently inflaming and excoriating the cuticle where applied, rendering the remedy much more tedious and painful than the disease. Although I have seen good effects from the application of Mr. Ring's ointment in several trials, still it is liable to the same objection; confident of this, and to obviate every inconvenience arising from irritation, was my sole motive for introducing the following formulas and plan of treatment, which I have invariably pursued with success beyond expectation.

No. 1. ℞. Calx Hydrar. Alb. ʒij. Cerufs. Acetat. Kali. pp. aa. gr. x. Ung. Simpl. ℥ij. M. cum Essent. Bergam. et Lavend. aa. gtt. xx. ft. Linm. omni noct. utend. h. s.

No. 2.

No. 2. ℞. Calx Hydrar. Alb. ʒj. Calomel pp. ℈j. Lac Sulph. ʒij. Eſſent. Lavend. & Bergam. aa. gtt. xxv. Adeps Suillæ ℥ij. M. applicetur omn. noct. h. s.

The preparation of ſulphur in this latter liniment is devoid of that diſagreeable ſmell which generally accompanies the other preparations of this kind, and ſeldom predominates: they are very neat applications; and it is rarely neceſſary to apply them more than two or three ſucceſſive times, before a perfect cure is obtained.

In cutaneous and ſcurfy eruptions of the head, and extending behind the ears, frequently obſerved in infants, where a thin ichor ſometimes pervades the cuticle, and almoſt excoriates when it is ſuffered to remain, I have found the following liniment, as a general application, of conſiderable utility, and ſeldom fail of effecting a radical cure.

℞. Creta pp. Calx Hydrar. Alb. aa. ℈j. Ceruſs. Acet. ℈j. Ung. Hydr. Nitrat. ʒij. * Unguent. Pice ℥ij. M.

Although I am extremely averſe to preciſe formulæ, yet cannot avoid recommending the above, the efficacy of which is ſurpriſing, when regularly applied; it abſorbs and corrects the acrid virus, whether from a venereal taint, or any other acrimonious eruption, ſo frequently to be obſerved in children, particularly on the head. It ſhould be applied every night, covering (at the ſame time) the parts with a bladder, or linen; and waſhed off in the morning with ſoap and water.

Alterative medicines are ſometimes neceſſary to be adminiſtered at the ſame time; and of thoſe, ſmall doſes, and ſuch preparations as are not likely to paſs by ſtool.

℞. Hydrar. cum Sulph. Magnes. Calc. aa. g v. M. ft. Pulvis.

Or a $\frac{1}{4}$ or $\frac{1}{2}$ a grain of calomel given every, or every other, night, may anſwer the purpoſe equally well, as the practitioner may think proper. The doſes muſt be varied, according to the age, conſtitution, &c. of the patients; and if acidity abounds in the firſt paſſages, ſhould be combined with abſorbents, ſuch as creta or magneſia, as the bowels may be more or leſs affected. It will be neceſſary to obſerve, that the bowels muſt be kept gently open.

*April* 28, 1802.

N. G.

---

* If this liniment is found too hard, a little olive oil may be added; or, if tar ointment ſhould be objected to, on account of its colour or ſmell, lard may be ſubſtituted.

